# Hip Reconstruction Osteotomy by Ilizarov Method as a Salvage Option for Abnormal Hip Joints

**DOI:** 10.1155/2014/835681

**Published:** 2014-05-06

**Authors:** Masood Umer, Haroon Rashid, Hafiz Muhammad Umer, Hasnain Raza

**Affiliations:** ^1^Department of Surgery, The Aga Khan University, Karachi 74800, Pakistan; ^2^The Aga Khan University, Karachi 74800, Pakistan

## Abstract

Hip joint instability can be secondary to congenital hip pathologies like developmental dysplasia (DDH) or acquired such as sequel of infective or neoplastic process. An unstable hip is usually associated with loss of bone from the proximal femur, proximal migration of the femur, lower-extremity length discrepancy, abnormal gait, and pain. In this case series of 37 patients coming to our institution between May 2005 and December 2011, we report our results in treatment of unstable hip joint by hip reconstruction osteotomy using the Ilizarov method and apparatus. This includes an acute valgus and extension osteotomy of the proximal femur combined with gradual varus and distraction (if required) for realignment and lengthening at a second, more distal, femoral osteotomy. 18 males and 19 females participated in the study. There were 17 patients with DDH, 12 with sequelae of septic arthritis, 2 with tuberculous arthritis, 4 with posttraumatic arthritis, and 2 with focal proximal femoral deficiency. Outcomes were evaluated by using Harris Hip Scoring system. At the mean follow-up of 37 months, Harris Hip Score had significantly improved in all patients. To conclude, illizarov hip reconstruction can successfully improve Trendelenburg's gait. It supports the pelvis and simultaneously restores knee alignment and corrects lower-extremity length discrepancy (LLD).

## 1. Introduction

Instability of hip joint in young adults is a challenging problem to treat. Chronic unstable hip joint is due to complication of infective processes, developmental dysplasia, and traumatic injury or neoplastic process [1]. Patients with such hips show loss of bone from proximal femur, proximal migration of femur, limb length discrepancy (LLD), complain of pain, abnormal gait, shorter step length, and decreased maximum adduction of hip and knee on the affected side [2].

Various treatment options include the following: arthrodesis, which is a good choice for unstable and painful hip but results in reduced hip range of motion (ROM) and adverse effects on lower back [3, 4], and total hip replacement (THR), which leads to increased ROM, pain relief, and restoration of LLD [5, 6]. However, in younger age groups, due to their active lifestyle, there is mechanical stress on the prosthesis which leads to implant loosening requiring a revision surgery within the next two decades [6–9].

Many authors have suggested proximal femoral valgus osteotomy for the treatment of unstable hip joints [10, 11]. Schanz emphasized that, in cases of proximal femoral instability, if the femur is angled in such a way that the upper fragment aligns with the sidewall of the pelvis and lower fragment aligns parallel to the axis of weight bearing, the instability and limping gait is improved because the stable position is reached. The lower femoral fragment must be extended backwards to decrease the pelvic tilt and lumbar lordosis [12]. Milch showed that the drawback of proximal femoral valgus osteotomy is the leg shortening and disturbance in mechanical axis of the leg [11]. Ilizarov modified this technique and performed a double-level osteotomy. In addition to proximal femoral valgus extension osteotomy, he introduced a distal femoral varus osteotomy which would lead to limb lengthening and correction of mechanical axis of the leg [11].

The purpose of this study was to present our clinical results and compare them with other studies using hip reconstruction osteotomy by Ilizarov method for unstable hip joint.

## 2. Methods

Our study included 37 patients who were all treated at our single institution between 2005 and 2011. There were 18 males and 19 females. Their mean age at the time of surgery was 23.30 (range: 15–35). They had various etiologies: 17 were diagnosed as neglected dysplastic dislocated hips (DDH), 12 were having complication of septic arthritis, 2 had history of tuberculous arthritis, 4 had posttraumatic arthritis, and 2 had proximal femoral focal deficiency.

Hip reconstruction osteotomy was given as treatment option to those patients who were skeletally mature with age range of 15–39 years, if they had chronically unstable hip joints, limping, pain, and positive Trendelenburg's sign.

Patients who were below 15 or above 40 years and had neuromuscular hip disorders, bilaterally unstable hip joints, and malignant diseases of bones were not included in the study.

Preoperative evaluation included detailed history and thorough general physical examination and local examination with particular emphasis on ROM, LLD, and Trendelenburg's sign and gait. All patients complained of limping, and 26 of them had moderate-to-severe pain while 11 of them had mild pain on movement. The pain was either in the thigh or in the lower back.

Modified Harris Hip Score was used to document pain and functional limitations due to restricted ROM [14, 15].

### 2.1. Radiographic Evaluation

**Figure 1:**
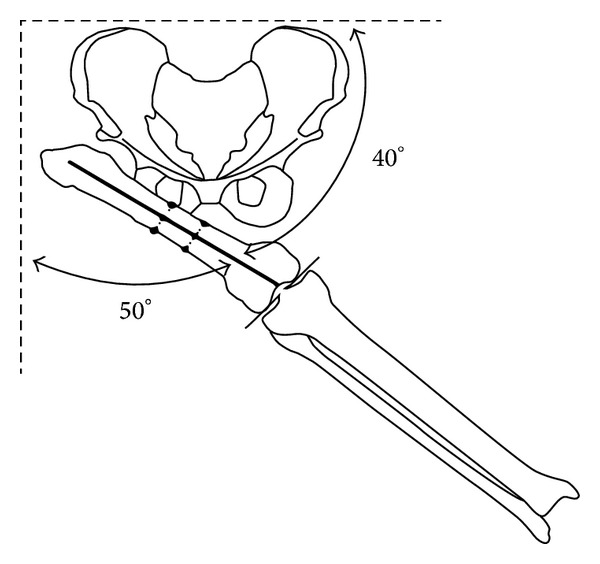
Level of proximal osteotomy. This figure was reproduced and modified from Dr. Paley's textbook* Paley D. Principles of Deformity Correction*, Springer, 2005, after his permission.

The radiographic assessment included anteroposterior (AP) view of the pelvis in neutral position and a maximum-adduction cross-legged anteroposterior radiograph of the pelvis (made with the patient supine with lower extremities adducted and the involved hip flexed and adducted over the top of the uninvolved hip); this determines the level of proximal femoral osteotomy and amount of acute abduction to be created in the proximal osteotomy (Figure 1).

Lower-extremity length discrepancy was calculated from a scanogram, which is AP view of pelvis and both lower extremities extending from hip to ankle joints on a single long film. It serves to document the presence of any deformities of the femur and tibia in the coronal plane in addition to the hip pathology. It also provides an estimate of limb length inequality, but any fixed flexion deformity of the hip should herald caution on the interpretation of these length measurements.

**Figure 2:**
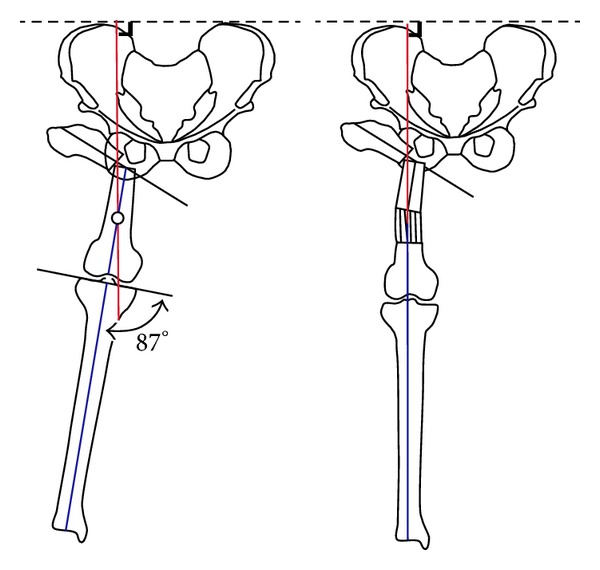
Level of distal osteotomy. This figure was reproduced and modified from Dr. Paley's textbook* Paley D. Principles of Deformity Correction*, Springer, 2005, after his permission.

The level of the second compensatory osteotomy was determined on a tracing paper of the pelvis and femur with the proximal femoral segment maximally adducted. The transecting point perpendicular to the pelvis, between the first osteotomy site and the mechanical axis of the distal femur, determines the level of the second osteotomy site (Figure 2).

### 2.2. Surgical Technique

All surgeries were performed together by two senior authors who are orthopedic consultants and specialist in Ilizarov methods of surgery. Patients were placed supine on the traction table. The proximal osteotomy site was identified and confirmed under fluoroscopy with ipsilateral hip in maximum adduction and it was the level at which the femoral shaft crossed the ischium. During skin preparation and draping, one of the authors assembled the fixator, which consists of a pelvic arch for holding the proximal femur segment, another arch or a 5/8 ring for the middle segment, and 2 rings for the distal segment. A motor is placed on the lateral side and hinges on the medial side between distal and middle segments of the assembly.

Three long 6 mm Schanz pins were inserted into proximal femur segment in the inclined position so that when the proximal femur is adducted after proximal osteotomy the pins should be parallel to the horizontal line of the pelvis from the lateral side. While inserting these pins, skin was stretched proximally to avoid tenting after the proximal femur was adducted after osteotomy. The pins were connected to an arch, which was kept perpendicular to the floor. For adjustment of extension, the arch was tilted accordingly.

The middle femoral fragment was fixed with three Schanz pins (6 mm) inserted in different planes but perpendicular to the mechanical axis of femur and attached with a complete ring or ring and arch block. In the distal femur, a 1.8 mm Ilizarov wire was inserted parallel to the knee joint line. It was tensioned and fixed to the distal ring of the assembly. Additional wires were inserted and fixed with the distal rings. Finally, the proximal and distal femoral osteotomies were done at the preplanned levels and confirmed for completeness in fluoroscope. Later on, the whole assembly was locked.

### 2.3. Postoperative Evaluation

All patients were assessed in outpatient clinics at regular intervals postoperatively for pain, ambulation, pin tract related complications like tightening of skin at pin insertion site or pin tract infections, and loosening of any wire or any component of the assembly. Patients began walking, and physical therapy was started within 48 hours.

Distraction at lateral side of distal osteotomy site was started on the seventh postoperative day for correction of the mechanical axis. Once the correct mechanical alignment was achieved, distraction was started for lengthening (if required). The amount of lengthening was decided from standing X-rays during follow-up examinations. Lengthening was continued until the horizontal axis of the pelvis became parallel to the ground in standing X-rays. The standard rate of distraction was 1 mm/day, but it was modified according to the rate of bone regeneration. In cases with slow bone formation, the rate was slowed to 0.5 mm/day and even stopped for few days.

**Figure 3:**
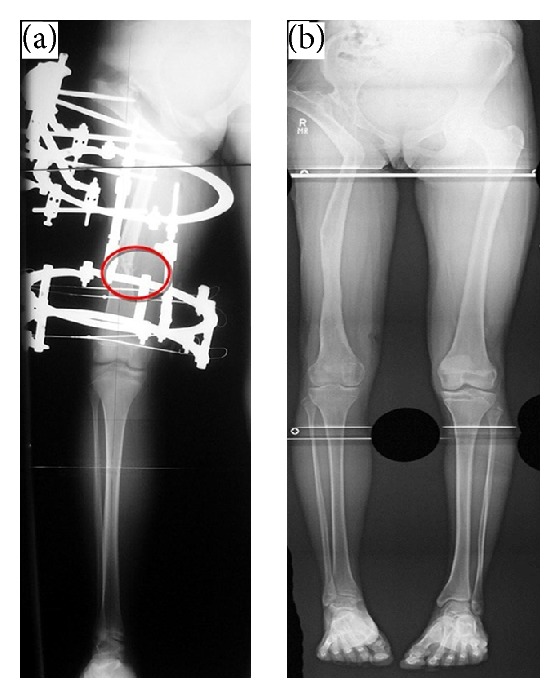
(a) Postoperative radiographs. (b) Postoperative radiograph after frame removal.

The mechanical axis was also assessed during postoperative follow-up, and adjustment of any mechanical axis deviation was performed as needed by adjustment of the external fixator. Clinical and radiographic follow-ups were done every 2 to 4 weeks until there was full consolidation and union of the proximal osteotomy and distraction callus at the distal osteotomy site (Figures 3(a) and 3(b)).

Removal of fixator was done in the clinic in most of the patients under local anesthesia and parenteral analgesia. Patients continued physical therapy after removal of external fixation until maximum function was regained.

Harris Hip Score was used at the outpatient follow-up for assessment of functional outcome.

**Figure 4:**
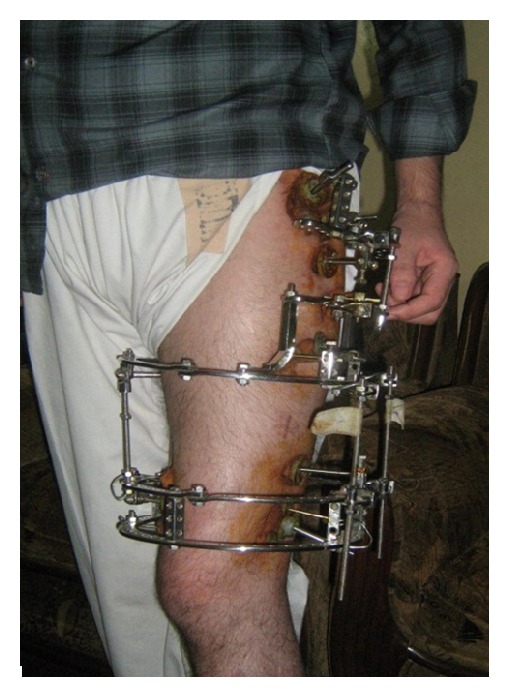
Patient after application of Ilizarov method.

**Figure 5:**
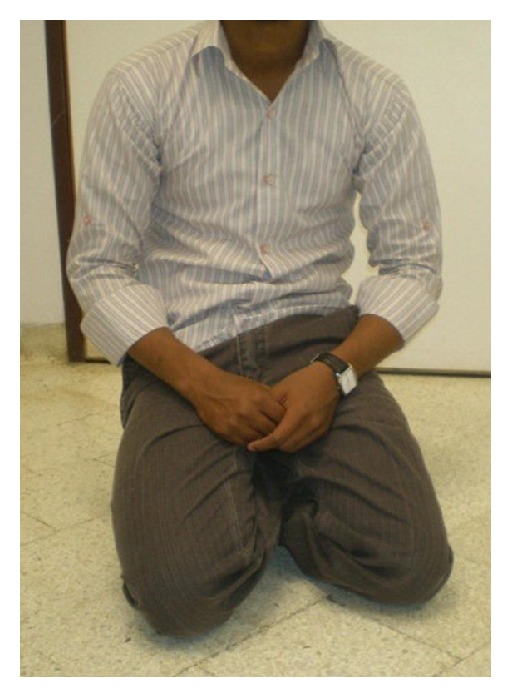
Follow-up after Ilizarov removal.

ERC/IRB approval was not required because this is a retrospective study (see Figures 4 and 5).

## 3. Statistical Analysis

The data was collected and revised. Subsequently, it was verified and then edited on a personal computer. The data was then analyzed using the SPSS software (SPSS Inc., Chicago, IL), and the results were represented in the form of means and standard deviation. *P* value was set to be significant at 0.05.

## 4. Results

All patients were evaluated for range of hip and knee motion, limb length, and Trendelenburg's sign. Treatment with pelvic support osteotomy improved the modified Harris Hip Score for all our patients. Harris Hip Score had improved from a preoperative mean of 46.4 (range: 3–78) to a postoperative mean of 87.7 (range: 72–98). Hip range of flexion and range of abduction had increased significantly postoperatively. Mean limb length discrepancy was significantly reduced postoperatively.

Table 1 compares the details of preoperative and postoperative data (Table 1).

The mean fixator interval was 9.9 months, ranging from 4 to 23 months, and the mean duration of follow-up was 29.6 months, with a minimum of 6 months and a maximum of 72 months. Pain on ambulation disappeared to varying degrees. Twenty-seven patients had no pain, while five had slight discomfort. Limping gait was also found to have improved. The limp of 22 patients was eliminated completely, while slight limp persisted in 15 of the patients.

Seventeen patients had no complications at all, while three had extension contracture at knee, 2 had a problem of nonunion, and 1 had fracture. Generally, patients were found to be satisfied because pain and limp had decreased (Table 2).

## 5. Discussion

The most important finding of the study was overall improvement in the functional status of patients as evident from Harris Hip Scores. The purpose of treating an unstable hip with hip reconstruction osteotomy and distal femoral lengthening is to reduce the limp, pain, and lumbar lordosis. It also increases the range of hip motion and equalizes the limb length [9].

Due to the advancements in surgical procedures and prosthesis design, total hip arthroplasty has become the first choice of treatment for unstable hips in young adults [9, 14]. Lai et al. [16] performed total hip arthroplasty on 22 women with unilateral congenitally unstable hips which resulted in significantly improved gait symmetry and efficiency and decreased LLD to within 2 cm. However, total hip arthroplasty has a high rate of complications in young adults which include early postoperative loosening and infections [17]. Also, revision of total hip arthroplasty in young patients with congenital dislocations is much more difficult than that in the standard procedure [18]. Total hip arthroplasty in hips that were infected previously was highly unsuccessful [19].

Different studies have shown that pelvic support osteotomy gives the best results when done in patients over the age of 15. Otherwise, patients will have to undergo the procedure again due to loss of proximal angulation with growth [9, 11]. In our study, mean age of patients was 23.3 years (range: 15–35), and during the follow-up there was no loss of correction. We have compared the results of our study with the previous ones done by different authors Table 3.

Optimal level of pelvic support is different according to different authors. Some suggested a more proximal level with the insertion of lesser trochanter into acetabulum, while others proposed a more distal osteotomy. Mahran et al. [12] favored a more distal osteotomy similar to the one done by Emara [10]. Osteotomy done at our institution was at the level of ischium on a fully adducted limb.

Equalization of lower limb discrepancy has an important role in maintaining gait mechanics. Without the use of shoe lift, pelvic drop cannot be avoided. Studies showed that an apparent lengthening would be achieved if distal fragment of femur was overabducted, but this would lead to genu valgum and excessive shear stress on the knee [2]. Limb lengthening of 5.63 (range: 0–11) was achieved during the follow-up in our study. There was no mechanical axis deviation and no genu valgum because distal osteotomy allows limb lengthening as well as correction of mechanical axis of the lower limb.

Trendelenburg's gait is another important problem in the hip instabilities. It actually leads to increased fatigue and pain on distance walking. The literature has shown that pelvic support osteotomy procedure is very helpful in removing Trendelenburg's gait. Arthrodesis is the only other method to eliminate this problem, but a large range of motion is lost in this method. In hip reconstruction osteotomy, acceptable and painless range of motion is preserved. The range of hip flexion and adduction decreases, while that of abduction and extension increases. Rozbruch et al. [14] reported a decrease in mean hip flexion of 26% and an increase in mean hip abduction by 20%.

Most patients in a third world country cannot afford THR. Doing cementless total hip replacement is five times more costly than doing a hip reconstruction osteotomy in our setup. Moreover, HRO is a lifetime procedure with no revisions anticipated as in the case of a total hip replacement. In our eastern culture, sitting on the floor and squatting is a day-to-day requirement in many activities of daily living. HRO provides adequate opportunity to observe these squatting requirements; hence, patient compliance is much better than a total hip replacement procedure. We recommend this procedure as a low cost salvage and culture friendly option for third world countries.

Tightening of quadriceps muscles because of femoral lengthening is known to cause knee stiffness postoperatively [20, 21]. This problem arises mostly due to noncompliance of patients with physiotherapy because of pain and heavy fixator apparatus. However, it can be resolved by doing aggressive post-fixator physiotherapy. Patients must also be taught to keep the pin tract areas clean to avoid pin tract infection.

Our experience also tells us that patients undergoing pelvic support osteotomy, despite significant improvement in their pain and limp, cannot walk for a long distance. They get fatigued.

This was a retrospective case series, which is the limitation of our study. There is a need for randomized control trials to get the better results, but it seems very difficult in our region due to cost issues.

Our study is the largest case series of this procedure reported in the literature. It will motivate and help the orthopedic surgeons to use hip reconstruction osteotomy as a viable option managing particular group of patients.

## 6. Conclusion

Hip reconstruction osteotomy is a viable option for treating those patients who are not good candidates for total hip replacement. It is a procedure of choice which simultaneously improves gait mechanics and limb length discrepancy with retention of joint range of motion. Knee stiffness and pin tract infections are common but avoidable complications of this procedure.

## Figures and Tables

**Table 1:** Shows summary of results.

	Pre-operative	Post-operative	*P*-value
Hip flexion (degrees)	Mean = 40.1	Mean = 120.0	.05
	(Range = 10–100)	(Range = 70–130)	
Hip abduction (degrees)	Mean = 7.7	Mean = 23.7	.02
	(Range = 0–30)	(Range = 13–30)	
Harris Hip score	Mean = 46.4	Mean = 87.7	.03
	(Range = 3–78)	(Range = 72–98)	
LLD (cm)	Mean = 6.6	Mean = 1.0	.0001
	(Range = 0–23)	(Range = 0–11)	

**Table 2:** Complications.

	*N* = 37
No complications	17
Major complications	
Extension contracture at knee	3
Non-union	2
Fracture	1
Minor complications (pin tract infection)	14

**Table 3:** Compares our results with other studies.

	Our study		Marimuthu et al.'s study [13]		Mahran et al.'s study [12]		Emara's study [10]		EL-Mowafi's study [2]	
Sample size	37		12		29		11		25	
	Pre-op	Post-op	Pre-op	Post-op	Pre-op	Post-op	Pre-op	Post-op	Pre-op	Post-op
LLD (Mean and Range in cm)	6.6 (0–23)	1.0 (0–11)	5.1 (2.5–6.8)	0.90 (0–3)	6.90 (4–11)	1.10 (0–3.5)	Length gained-4.9 cm		5.3 (3–8)	0.00 (0-0)
							Range (3–7)		
Harris Hip Score (Mean and Range in cm)	47 (3–78)	87 (72–98)	44 (14–73)	70 (60–86)	—		43 (31–50)	71 (65–80)	55 (40–78)	81 (65–90)
ROM Flexion (Mean and range in degrees)	40.1 (10–100)	120.0 (70–130)	88.3 (70–120)	70.4 (45–105)	87.7 (30–40)	72.2 (30–120)	90.0 (80–120)	124 (100–140)	90 (40–120)	127 (100–140)
ROM Abduction (Mean and range in degrees)	7.7 (0–30)	23.7 (13–30)	12.1 (0–25)	22.5 (15–35)	37.7 (10–70)	45.7 (15–75)	8.0 (0–15)	22.0 (10–30)	8.0 (0–15)	30.0 (10–50)
